# Effect of Beverage Consumption Frequency on DMFT Index among Iranian Adult Population: An AZAR Cohort Study

**DOI:** 10.1155/2022/9142651

**Published:** 2022-05-27

**Authors:** Mahdi Hadilou, Mohammad Hossein Somi, Elnaz Faramarzi, Leila Nikniaz

**Affiliations:** ^1^Student Research Committee, Faculty of Dentistry, Tabriz University of Medical Sciences, Tabriz, Iran; ^2^Liver and Gastrointestinal Diseases Research Center, Tabriz University of Medical Sciences, Tabriz, Iran; ^3^Tabriz Health Services Management Research Center, Faculty of Management and Medical Informatics, Tabriz University of Medical Sciences, Tabriz, Iran

## Abstract

**Background:**

Given the effect of oral and dental condition on emotional and physical health and the different effects of beverage consumption on decayed, missing, and filled teeth (DMFT) as one of the indicators of oral health in different populations, this study aimed to investigate the effect of beverage consumption and its frequency on DMFT among Iranian adult population.

**Materials and Methods:**

The data of this cross-sectional study were obtained from AZAR (Azerbaijan) cohort, conducted on 15,006 adults aged 35 to 70 years old in Shabestar County, Iran. Demographic and nutritional data were collected by nutritionists through the “Food Frequency Questionnaire,” and DMFT index by general practitioners trained by a dentist according to the World Health Organization (WHO) guidelines. Beverages including milk, doogh, natural fruit juice, black tea, nonalcoholic beer, coffee, sugar-sweetened beverages, and artificial fruit juice were studied.

**Results:**

A total of 14,966 adults with the mean age of 49.64 ± 9.28 were enrolled. The participants' mean DMFT value was 21.27 ± 8.95. Weekly consumption of tea and coffee beverages resulted in 13% (95% confidence interval: 3% to 22%) lower DMFT compared to daily consumption (*p*=0.01). However, there was no association between DMFT and other beverages.

**Conclusion:**

The higher DMFT values with the daily consumption of tea and coffee beverages showed that a part of adults' oral health is affected by beverages; therefore, this should be considered by healthcare authorities in order to manage carries development in the communities.

## 1. Introduction

Oral diseases impose extensive negative effects on patients, their families, and the health care system. The financial burden of neglected oral health on societies is vastly underestimated around the world [1]. It has been shown that oral diseases such as dental caries, tooth loss (edentulism), periapical and periodontal complications have a bidirectional relationship with diet alterations, systemic diseases, emotional discomfort, and aesthetic problems, which highlights the importance of achieving and maintaining appropriate oral hygiene [2–5].

Despite a decreasing trend in dental caries, they are still among the most prevalent diseases worldwide, and have involved most adult communities, including both low and high income [6]. To quantify dental health status, the Decayed, Missing, and Filled Teeth (DMFT) index has been introduced as a representative of oral health by Klein and Palmer in 1938. DMFT index is composed of the sum of decayed, missing, and filled teeth. Regardless of its limitations, it still is an acceptable epidemiological index in dentistry that represents the oral and dental health condition of any given society or individual. It also is identified as the “Caries Index” [7].

Factors affecting dental caries' incidence and prevalence include lifestyle, genetics, and nutrition [8]. Also, consumption of different beverages and its frequency has a major role in preventing or accelerating dental caries. However, disparate opinions still exist to date [9].

According to the literature, a dose-response association has been reported between DMFT and consumption of sugar-sweetened soft beverages (SSBs) in the Finnish adult population [10]. However, only a weak association was observed in another study with the same population [11]. According to a systematic review with 36 included cohort and cross-sectional studies by Burt and Pai, arguments about the role of SSBs on dental caries exist in various populations. They stated that in the modern age, due to the fluoride supplementation of beverages, the conventional association between sugar consumption and dental caries development has been much weakened [12].

In the case of milk consumption, Park and Jang introduced milk as an anticariogenic beverage if consumed after sugar with the ability to buffer saliva by hampering the sugar fermentation [13]. On the contrary, Johansson et al. found no association between DMFS and milk consumption through a large-scale cohort [14]. Other studies have taken a more neutral stand, stating that the milk is less cariogenic than other beverages but not an anticariogenic agent [15, 16].

To date, a number of studies have been conducted to evaluate beverage consumption frequencies and their effects on the DMFT in different countries [9, 10, 17]. The consumption pattern of various beverages could be different among populations, and environmental factors like temperature and humidity might also alter these patterns [18–21]. Furthermore, due to the scarcity of a large and nationwide data on beverages and dental caries concerning Iranian adults, this study aimed to evaluate the effect of beverage consumption frequency on the DMFT index in the Iranian adult population.

## 2. Methods

### 2.1. Study Settings

This cross-sectional study used the data obtained from the AZAR (Azerbaijan) cohort (a subset of The Prospective Epidemiological Research Studies of the Iranian adults (PERSIAN) cohort [22]). AZAR cohort commenced in October 2014 and ended in January 2017. It contained a large sample size of 15,006 adults with the age range of 35 to 70 years in Shabestar city, the capital city of Shabestar County, East Azerbaijan province, located in the northwest of Iran [23]. The population of Shabestar county was composed of 25,663 individuals, in 4824 households at the 2011 census [24]. The study protocol has been approved by the Ethics Committee of Tabriz University of Medical Sciences (code: IR.TBZMED.REC.1400.973). Written informed consent was gathered from all of the participants, and they were free to leave the study any time they wanted. All eligible adults aged 35 to 70 years were invited to participate. The aim and steps of the study were completely explained to the participants, then anyone who filled the informed consent was included. The design, execution phases, and eligibility criteria of the AZAR cohort are explained in detail in previous published papers [22, 23].

### 2.2. Data Gathering

Three forms were provided for the participants. First, the “General information” form that covered sociodemographic characteristics. The second was the “Oral health” form, including five questions about oral and dental health. The data obtained from two questions in this section were used to conduct analyses. The first was “Do you floss your teeth? (1). Yes (2). No; If yes, How many times per day?,” the second was related to the DMFT value which was completed by a general practitioner who was trained by a skilled dentist according to the World Health Organization (WHO) Oral Health Surveys Basic Methods [25] to properly identify the DMFT in an individual. For dental examination, the patient sat on a chair, and the trained general practitioner recorded DMFT index with headlight, intraoral mirror, and an explorer probe. Before dental examination, patients were suggested to wash their teeth to provide a clear view, if not possible, the examiner used a piece of sterile gauze to clean teeth surfaces to have a better diagnosis.

**Figure 1:**
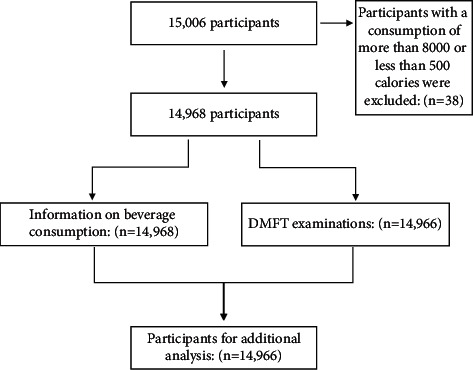
Flow chart for study stages and participant enrollment.

The third form entitled “Food Frequency Questionnaire” contained questions about consumption frequencies of different beverages in the last day, week, month, and year. Also, the amount of beverages consumed each time was recorded. This form was filled out through interviews by trained nutritionists. To obtain a reliable and consistent answer, a booklet was designed containing pictures of common beverages with different proportion sizes. Also, there were pictures of common cups and glasses to standardize the obtained results. Participants with an intake of less than 500 Calories and more than 8000 Calories (38 participants) were excluded. A diagram for study stages and participant enrollment is available as Figure 1.

To have a simple understanding and interpretation of the results, beverage consumption frequencies were divided into four groups, including: “never” (no history of drinking a specific beverage in the last year), “occasionally” (less than once a week), “weekly” (almost once a week), and “Daily” (almost once a day). The data related to eight beverages including milk, doogh, natural fruit juice, black tea, soft beverages, beer beverages (non-alcoholic), coffee beverages, and artificial fruit juice were recorded. These beverages were divided into four groups. The first and second groups were “Milk” and “Doogh.” The third group was “Tea and coffee beverages,” including black tea, coffee, and Nescafé. The fourth was “Natural fruit juice and nonalcoholic beer,” and the fifth was “Sugar-sweetened soft beverages” that included artificially sweetened beverages, chocolate milk, and soda. These beverages could be consumed solely, with additives or other beverages.

### 2.3. Statistical Analysis

The chi-square test was used to compare the qualitative variables. Numbers and percentages were reported for qualitative variables, and DMFT was reported as mean and standard deviation (mean ± SD). Owing to the normal distribution of the data, one-way ANOVA test was used to compare DMFT and beverage consumption frequency. Negative binomial regression was performed in two models. The first model was not adjusted, and the second model was adjusted for gender (male and female), socioeconomic status (poorest to richest in five quantiles), age, and brushing (at least once a day, less than once a day). The socioeconomic status was determined via multiple correspondence analysis (MCA). Each individual's socioeconomic status was computed by assessing their possession of a variety of durable assets (e.g., dishwasher, car, and TV), house condition (e.g., the number of rooms and type of ownership), and educational levels (illiterate, primary, diploma, and college). Therefore, the participants were divided into five socioeconomic status quintiles, ranging from the lowest to the highest ones (1st to 5th quintile, respectively).

The significance level was considered *p* < 0.05. SPSS version 17 for Windows was used to perform data analyses.

## 3. Results

Data of 14,966 participants with the mean age of 49.64 ± 9.28, including 6678 men (44.7%) with the mean age of 50.14 ± 9.23 and 8290 women (55.3%) with the mean age of 49.27 ± 9.26 were analyzed in this study. The male/female ratio was 0.8. A number of 2486 participants (16.6%) were illiterate, 5852 (39.1%) had primary school degree, 5328 (35.6%) had diploma, and 1302 (8.7%) had college degree. Furthermore, 38.1% of the participants were in 35 to 45 age group, 33.3% in 46 to 55, 22.8% in 56 to 65, and 5.8% in 66 to 70. The total mean DMFT value was 21.26 ± 8.95.

Table 1 shows the frequency of beverage consumption in the total participants. The most frequently used beverages on a daily basis were black tea and coffee beverages (96.3% of the participants), followed by milk (36.0%), and SSBs (3.9%). Only 1.1% of participants had never consumed tea in 1 year. The consumption of tea and coffee beverages (*p* < 0.001), and milk derivates (*p* < 0.001) were equal between genders. However, men consumed SSBs (*p* < 0.001), and natural fruit juice and nonalcoholic beer (*p* < 0.001) more than women.

The most popular beverages consumed occasionally were natural fruit juice and nonalcoholic beer (48.0%), followed by SSBs (41.1%), and doogh (36.3%). Only 2.1% of the participants had not had milk in the past year. Furthermore, 55.5% of participants had weekly, and 36.0% had daily milk consumption.

According to the univariate analysis (Table 2), there was an association between all four beverage groups and DMFT (*p* < 0.01 for milk derivatives, and *p* < 0.001 for tea and coffee beverages, SSBs, doogh, natural fruit juice, and nonalcoholic beer).

The results of negative binomial regression are presented in Table 3. According to Model 1, adults who consumed black tea and coffee beverages weekly had 14% (95% CI: 4%-23%) lower DMFT values compared to daily consumption (*p* < 0.01). This association was also confirmed in Model 2 presenting 13% (95% CI: 3%-22%) lower DMFT values in weekly consumption of black tea and coffee beverages compared to daily consumption (*p*=0.01). In other words, the daily consumption of black tea and coffee beverages resulted in 14.9% greater DMFT values compared to weekly consumption. Furthermore, DMFT was not associated with other beverage groups.

## 4. Discussion

Consumption of different beverages and its frequency has a major role in preventing or accelerating dental caries. However, major arguments still exist in the literature [9]. This study aimed to evaluate the effect of beverage consumption on DMFT among Iranian adult population with the leading outcome that the tea and coffee beverages increased the DMFT, whereas other beverage groups had no substantial effect.

Based on the results of this study, DMFT had no association with natural fruit juice and SSB consumption. In a 4-year prospective study among Finnish adults aged more than 30 by Bernabe et al., it was reported that the high SSB consumption increased DMFT up to 31% to 33% [10]. On the contrary, in another study investigating the same database as Bernabe et al., only a weak association was observed [11]. Bernabe et al. suggested that these controversies could be explained by the different methods that sugar consumption frequencies were operationalized [10]. Since both frequency and amount of sugar intake are major risk factors for dental caries [26], it should be noted that the daily SSB consumption in this study's participants (3.9%) was much lower than Bernabe et al. reports (47%) [10]. Thereby, the small exposure group might be the reason that prevented this study to find a relationship between SSB consumption and DMFT.

In a systematic review by Burt and Pai investigating the association of sugar consumption and caries risk in the countries with moderate to high fluoride supplementation that included 36 high-quality cohort and cross-sectional studies; 18 papers reported weak association, 16 found a moderate, and just 2 reported a strong relationship. They suggested that in the modern age, due to the fluoride supplementation of beverages, the conventional association between sugar consumption and dental caries has been much weakened [12]. Overall fluoride exposure from water [27, 28] and food [29] in the population of this study was in line with the U.S. Public Health Service recommendations to prevent dental caries [30]. Therefore, this could be a possible reason that led to dampening of the potential association between beverages and DMFT.

Based on the results of this study, DMFT was not associated with milk consumption. Regardless of the indirect role of the milk as one of the ways of supplying calcium to the body, and the role of calcium in tooth structure growth and development [31], there are contradictory opinions about the benefit of direct contact of milk with teeth in dental caries development. Some studies state that milk contains a sugar called “lactose” which lowers the bacterial plaque pH providing a suitable environment for caries' progression [15]. However, other studies state that it also contains bioactive components and proteins that impede adhesion and metabolism of inherent dental plaque bacteria [32–34].

Johansson et al. performed an experimental study alongside the cross-sectional analysis of a large-scale cohort data, studying 31,571 individuals. They found an inverse association between milk consumption and bacterial load of dental plaque in the experimental part. However, it was inconsistent with the results obtained from the cohort data analysis which showed no association between milk consumption and DMFS index. They suggested that due to the complex and multifactorial nature of dental caries, their progression could have been modulated by different consumption patterns of food groups along with milk [14].

A study by Adegboye et al. [35] on 432 Danish adults reported an inverse relation between milk and dental caries. Other studies have stated that milk is less cariogenic than other beverages but not anticariogenic [15, 16]. It should be noted that due to the complexity of measuring the effect of various additives, artificial colors, and sweeteners added to the beverages by manufacturers or consumed alongside the beverages by participants, which can dampen the potential anticaries effect of milk in the observational population-based studies, this study should be followed by large trials controlling the effect of these confounding factors.

The results showed that weekly consumption of tea and coffee beverages caused 13% lower DMFT values compared to daily consumption (*p*=0.01). In other words, daily consumption of this beverage group resulted in 14.9% more DMFT values compared to weekly consumption. Furthermore, 96.3% of participants consumed tea and coffee beverages daily with high frequency each day, which is in line with two published studies conducted in Iran [9, 36]. Furthermore, it should be considered that most of the tea drinkers in Iranian population, traditionally drink hot black tea and chew a significant amount of sugar cubes while drinking it [36]. This might have affected the results because sugar is a leading cause of dental caries [37, 38]. This issue also might have manipulated the effect of all beverage groups in this study.

Adding significant amounts of sugar to the tea and coffee beverages, and its relationship with higher rate of root caries occurrence are also observed in other countries [39]. This issue could be solved by using smaller cubes of sugar or consuming healthy alternatives like raisins which contain substances that inhibit *Streptococcus* Mutans' growth, the main bacteria involved in dental caries development [40].

### 4.1. Strengths and Limitations

Including a large-scale sample was the strength of this study. Nevertheless, it had some limitations. One was its cross-sectional design. In cases of assessing DMFT, prospective studies (with the advantage of follow-up measurements) that compare the amount of change in DMFT and beverage consumption in a specific time period can show the associations and causality better than cross-sectional studies.

Furthermore, the data obtained from the AZAR cohort had been gathered in a large-scale area throughout the county, including rural and urban inhabitations. Therefore, there may have not always been an available dentist in every health center. Thus, the oral examinations were performed by the resident general practitioners trained by a specialist dentist according to WHO guidelines. This also might have affected the results.

Ultimately, recall bias could be a limitation due to the patient-dependent reporting of beverage consumption frequency data that were collected from participants in a face-to-face interview.

## 5. Conclusion

This study showed that the weekly consumption of tea and coffee beverages resulted in 13% lower DMFT values compared to daily consumption. This inverse association indicated that a part of adults' oral health is affected by beverages. Therefore, this should be considered by healthcare authorities to manage caries development in communities. Furthermore, large trials with strong control of the confounding factors are expected in order to address the diversity between the results of in-vitro, experimental, and observational studies.

## Figures and Tables

**Table 1:** Beverage consumption status in participants.

Group	Gender	Never *N* (%)	Occasionally *N* (%)	Weekly *N* (%)	Daily *N* (%)	*p* value^a^
Milk derivatives	Male	598 (9.0)	1689 (25.3)	2789 (41.8)	1602 (24.0)	<0.001^*∗*^
	Female	1084 (13.1)	2040 (24.6)	2971 (35.8)	2195 (26.5)	
	Total	1682 (11.2)	3729 (24.9)	5760 (38.5)	3797 (25.4)	

Doogh	Male	497 (7.4)	2238 (33.5)	3153 (47.2)	790 (11.8)	<0.001^*∗*^
	Female	876 (10.6)	3189 (38.5)	3260 (39.3)	965 (11.6)	
	Total	1373 (9.2)	5427 (36.3)	6413 (42.8)	1755 (11.7)	

Tea and coffee beverages	Male	48 (0.7)	18 (0.3)	94 (1.4)	6518 (97.6)	<0.001^*∗*^
	Female	79 (1.0)	33 (0.4)	275 (3.3)	7901 (95.3)	
	Total	127 (0.8)	51 (0.3)	369 (2.5)	14419 (96.3)	

Natural fruit juice and nonalcoholic beer	Male	1222 (18.3)	3247 (48.6)	2103 (31.5)	106 (1.6)	<0.001^*∗*^
	Female	2823 (34.1)	3931 (47.4)	1479 (17.8)	57 (0.7)	
	Total	4045 (27.0)	7178 (48.0)	3582 (23.9)	163 (1.1)	

Sugar-sweetened soft beverages	Male	795 (11.9)	2536 (38.0)	3003 (45.0)	344 (5.2)	<0.001^*∗*^
	Female	1750 (21.1)	3617 (43.6)	2676 (32.3)	247 (3.0)	
	Total	2545 (17.0)	6153 (41.1)	5679 (37.9)	591 (3.9)	

^a^Chi-square test, ^*∗*^statistically significant.

**Table 2:** DMFT index by beverage consumption frequencies.

		DMFT Mean ± SD	*p* value^a^
Milk derivatives	Never	20.89 ± 9.11	<0.01^*∗*^
	Occasionally	21.40 ± 9.01	
	Weekly	20.91 ± 8.88	
	Daily	21.83 ± 8.88	

Doogh	Never	22.31 ± 8.69	<0.001^*∗*^
	Occasionally	21.27 ± 8.90	
	Weekly	21.02 ± 8.97	
	Daily	21.29 ± 9.14	

Tea and coffee beverages	Never	18.74 ± 9.14	<0.001^*∗*^
	Occasionally	18.15 ± 9.00	
	Weekly	18.47 ± 9.16	
	Daily	21.37 ± 8.92	

Natural fruit juice and nonalcoholic beer	Never	22.05 ± 9.02	<0.001^*∗*^
	Occasionally	20.95 ± 8.93	
	Weekly	21.00 ± 8.84	
	Daily	21.51 ± 8.94	

Sugar-sweetened soft beverages	Never	22.14 ± 8.97	<0.001^*∗*^
	Occasionally	20.83 ± 8.97	
	Weekly	21.37 ± 8.88	
	Daily	20.93 ± 8.92	

^a^One-way ANOVA test, ^*∗*^statistically significant.

**Table 3:** Modeling DMFT based on negative binominal regression model^a^.

		Model 1^b^ RR (95% CI)	*p*-value	Model 2^c^ IRR (95% CI)	*p*-value
Milk derivatives	Never	0.95 (0.90–1.01)	0.14	0.99 (0.93–1.05)	0.79
	Occasionally	0.98 (0.93–1.02)	0.40	1.00 (0.95–1.04)	0.99
	Weekly	0.95 (0.91–0.99)	0.05	0.98 (0.94–1.03)	0.58

Doogh	Never	1.04 (0.97–1.12)	0.20	1.03 (0.96–1.11)	0.37
	Occasionally	0.99 (0.94–1.05)	0.98	0.99 (0.93–1.04)	0.77
	Weekly	0.98 (0.93–1.04)	0.65	0.99 (0.93–1.04)	0.72

Tea and coffee beverages	Never	0.88 (0.73–1.04)	0.15	0.87 (0.73–1.05)	0.15
	Occasionally	0.85 (0.64–1.12)	0.26	0.86 (0.65–1.14)	0.30
	Weekly	0.86 (0.77–0.96)	<0.01^*∗*^	0.87 (0.78–0.97)	0.01^*∗*^

Natural fruit juice and nonalcoholic beer	Never	1.02 (0.87–1.20)	0.76	0.97 (0.82–1.14)	0.74
	Occasionally	0.97 (0.83–1.14)	0.74	0.96 (0.82–1.13)	0.66
	Weekly	0.97 (0.83–1.14)	0.76	0.98 (0.83–1.15)	0.85

Sugar-sweetened soft beverages	Never	1.05 (0.96–1.16)	0.23	0.92 (0.84–1.01)	0.10
	Occasionally	0.99 (0.91–1.08)	0.92	0.93 (0.85–1.01)	0.10
	Weekly	1.02 (0.93–1.11)	0.63	0.98 (0.90–1.07)	0.69

^a^“Daily” consumption frequency was considered as the reference variable, ^b^Model 1 was not adjusted, ^c^Model 2 was adjusted for gender, age, brushing, and socioeconomic status, ^*∗*^statistically significant.

## Data Availability

The data used and/or analyzed during the current study are available from the corresponding authors.
